# Skeletal Fiber Type in Muscle Pain and Dysfunction

**DOI:** 10.3390/biomedicines14040794

**Published:** 2026-03-31

**Authors:** Maria Lopes Cardia, Bruno Daniel Carneiro, Isaura Tavares, Daniel Humberto Pozza

**Affiliations:** 1 Unit of Experimental Biology, Department of Biomedicine, Faculty of Medicine, University of Porto, 4200-319 Porto, Portugal; up202006288@edu.med.up.pt (M.L.C.); bcarneiro@med.up.pt (B.D.C.); isatav@med.up.pt (I.T.); 2 Rheumatology Service, Unidade Local de Saúde do Alto Minho, Hospital Conde de Bertiandos, 4990-078 Ponte de Lima, Portugal; 3 Institute for Research and Innovation in Health and IBMC, University of Porto, 4200-135 Porto, Portugal

**Keywords:** muscle fibers, skeletal, muscle fibers, slow-twitch, muscle fibers, fast-twitch, musculoskeletal pain, chronic pain, delayed onset muscle soreness, satellite cells, skeletal muscle, muscle regeneration

## Abstract

Different types of skeletal muscle fibers display marked heterogeneity in metabolic, mechanical, and regenerative properties. However, their role in chronic musculoskeletal pain remains insufficiently integrated into clinical models. Chronic pain is associated with altered neuromuscular control, prolonged low-level activation, and reduced recruitment of high-threshold motor units. These factors may promote fiber type-specific remodeling. This narrative review critically synthesizes current evidence on the relationship between musculoskeletal pain and muscle fiber types. The focus was on metabolic vulnerability, mechanical susceptibility, and regenerative capacity. A structured literature search was conducted in PubMed, Scopus, and Web of Science, focused on human studies and key translational models. Chronic musculoskeletal pain is characterized by acquired fiber type-specific adaptations rather than a fixed unfavorable profile. In chronic pain scenarios, Type I fibers present features of chronic overload, including hypertrophy with insufficient capillarization and increased satellite cell activity. Type II fibers exhibit relative disuse, atrophy, and reduced satellite cell content, resembling accelerated muscle aging. Symptom duration, neuromuscular control strategies, and task-specific loading patterns modulate these adaptations, with interindividual variation. Muscle dysfunction in chronic pain reflects maladaptive but potentially reversible neuromuscular and histological plasticity. These findings indicate that rehabilitation strategies should be individualized, involving context-specific exercise strategies to restore muscle structure, function, and regenerative potential in chronic musculoskeletal conditions.

## 1. Introduction

Skeletal muscle fibers display marked heterogeneity in contractile behavior and metabolism, traditionally divided into slow-twitch (Type I) and fast-twitch (Type II) fibers, with the latter further subdivided into Type IIA or IIX (IIB in small mammals). Fast-twitch fibers are predominantly glycolytic and optimized for phasic, high-power contractions. In contrast, slow-twitch fibers are rich in myoglobin and oxidative enzymes, supporting sustained, low-intensity activity [1,2,3,4].

Histochemical myosin adenosine triphosphatase (ATPase) and oxidative enzyme staining led to the classic classification into slow oxidative (Type I), fast oxidative-glycolytic (Type IIA), and fast glycolytic (Type IIX) fibers. Both Type IIA and IIX (IIB) fibers possess high glycolytic capacity, but they differ in oxidative enzyme content, which underlies their distinct fatigue profiles and metabolic phenotypes [1,2,3,5].

**Figure 1:**
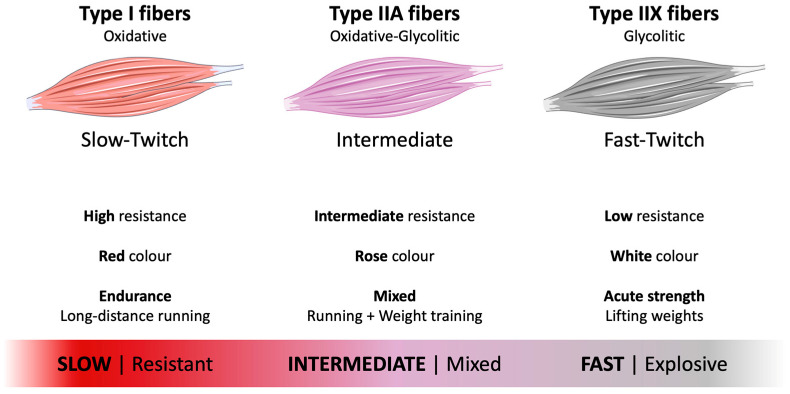
Overview of skeletal muscle fibers. Parts of this figure were drawn using pictures from Servier Medical Art by Servier (https://smart.servier.com, accessed on 5 January 2026), which is licensed under Attribution 4.0 International (Creative Commons CC BY 4.0).

In humans, the modern classification of skeletal muscle fibers is based on myosin heavy chain (MHC) isoforms. Type I fibers express MHC-I, Type IIA express MHC-IIA, and Type IIX express MHC-IIX, whereas adult human skeletal muscle does not express the “true” MHC-IIB isoform found in smaller mammals such as rodents [5,6]. Genetic and electrophoretic analyses from the 1990s onward confirmed that fibers historically mislabeled IIB in humans actually express MHC-IIX, while genuine MHC-IIB expression is restricted to certain non-human species [5,6]. Figure 1 shows a summary of skeletal muscle fiber types.

Under equivalent tension, fast fibers exhibit higher adenosine triphosphate (ATP) consumption than slow-twitch fibers, reflecting their role in rapid, powerful contractions at the cost of energetic efficiency. The spectrum of myosin isoforms determines shortening velocity and power output: slow myosin supports low-velocity, energy-saving contractions, whereas fast isoforms (IIA, IIX) allow for higher velocity and peak power but at greater tension cost [7,8,9].

During intense contraction, ATP turnover can increase up to 1000-fold above resting levels [10], sustained initially by phosphagen systems and then by glycolysis and mitochondrial oxidative phosphorylation. Slow-twitch fibers are better equipped for long-term ATP regeneration via oxidative metabolism and fatty acid utilization, whereas fast-twitch fibers rely more heavily on glycolytic pathways and glycogen stores, making them more powerful but less fatigue-resistant [7,8,9].

At the whole-muscle level, fatigue is expressed as a decline in force, velocity, and power. Muscles enriched in Type II fibers are typically stronger but fatigue faster than those with a higher proportion of Type I fibers [11,12]. Individuals with a predominantly fast-twitch typology exhibit more pronounced high-intensity exercise-induced fatigue and delayed recovery (up to several hours), compared with those with a slow-twitch-dominant profile, who will recover within minutes [11].

Muscle damage can occur when mechanical stress exceeds the structural tolerance of muscle fibers, particularly during unaccustomed or high-force eccentric contractions. Such contractions produce sarcomere overstretching, Z-line streaming, and cytoskeleton disruption, leading to loss of force and activation of inflammatory pathways [13,14]. As structural integrity deteriorates, damage-associated molecular patterns (DAMPs) are exposed, prompting the activation of resident macrophages, mast cells and the subsequent recruitment of circulating leukocytes to the injury site. This immune infiltration amplifies the release of pro-inflammatory cytokines, which not only contribute to debris clearance but also propagate the broader muscle-damage response through enhanced inflammation and oxidative stress [15,16,17]. Severe eccentric loads can induce substantial structural damage and prolonged recovery lasting more than a week, whereas typical resistance-type training elicits only mild myofibrillar disruptions with recovery over a few days [13,18].

Skeletal muscle regeneration depends on satellite cells: quiescent stem cells located between the basal lamina and sarcolemma that activate in response to growth or injury [19,20]. Following muscle damage, satellite cells exit quiescence, proliferate, and differentiate to repair or replace myofibers, a process strongly influenced by both intrinsic factors and signals from the surrounding niche [21,22]. Satellite cell activation increases markedly after resistance exercise, with pair box 7 protein (Pax7) positive cell content rising 24–72 h post-exercise, supporting early phases of muscle repair. Pax 7 protein is essential for maintaining the regenerative capacity of satellite cells, with the coordinated activity of these cells being essential for restoring muscle structure and function after injury [23,24].

Aging is associated with fiber type-specific vulnerability, with a marked decline in Type II fiber size and associated satellite cell content, while Type I fiber size and satellite cell numbers are relatively preserved [19,20]. These age-related changes can be reversed to some extent. In fact, resistance-type training in older adults can increase Type II fiber cross-sectional area and replenish the satellite cell pool, thereby counteracting selective Type II atrophy and enhancing regenerative capacity [25]. In women with chronic trapezius myalgia, specific strength training induces robust expansion of the satellite cell pool and macrophage content in the painful muscle, with satellite cell content per fiber increasing by more than 150% in Type II fibers and by over 50% in Type I fibers [25].

Chronic musculoskeletal pain (CMP), defined in the latest version of the International Classification of Diseases (ICD-11) as pain arising from bone, joint, muscle, or related soft tissues lasting longer than three months, is highly prevalent and often disabling [26]. In 2019, an estimated 2.41 billion people had conditions amenable to rehabilitation, with low back pain representing the most prevalent condition in most countries and contributing substantially to years lived with disability, particularly in middle-aged and older adults and in women [27]. Approximately 1.7 billion prevalent cases of musculoskeletal disorders (MSDs) were reported in 2021, nearly double the number in 1990, with projections exceeding 2.1 billion cases by 2035, despite declining age-standardized mortality rates [28,29]. MSD prevalence rises from childhood to older age, peaking in the 55-year to 89-year range. It is consistently higher in females than males, and is more common in individuals with low and medium socioeconomic status compared with those with higher socioeconomic status [29].

Sex-related differences in muscle fiber morphology and composition are well established. While Type I fibers tend to show similar diameters between men and women, Type II fibers are typically smaller in women, reflecting a sex-specific structural and functional characteristics of fast-twitch fibers. Moreover, men generally exhibit larger cross-sectional areas of both Type I and Type II fibers, as well as a greater proportional area and distribution of Type II fibers. In contrast, women display a higher proportional area and distribution of Type I fibers. These differences persist across age groups, physical activity levels, muscle groups, and analytical techniques, indicating that the divergence in muscle fiber characteristics reflects inherent biological sex differences rather than solely environmental or training-related influences [4,30]. Animal models further indicate sexually dimorphic MHC expression, with males exhibiting a higher proportion of IIB fibers and females a relative predominance of IIA-expressing cells in certain muscles, underscoring hormonal and genetic influences on fiber type distribution [31].

Occupational loading patterns also appear to modulate fiber morphology and pain risk [32,33,34]. In workers exposed to repetitive, low-level static tasks, such as cleaners, a reduction in Type II fiber cross-sectional area in the trapezius has been observed, whereas teachers show larger Type II fibers, possibly reflecting different recruitment patterns of slow and fast units during daily tasks [32,33]. According to the size principle, low-threshold Type I motor units are recruited first and remain active during low-force static work. This formed the basis of the Cinderella hypothesis, which proposes that low-threshold Type I motor units are recruited early during muscle activity and remain continuously active during prolonged low-intensity tasks. Because they are rarely allowed to rest, these fibers may accumulate metabolic stress and structural damage over time, leading to selective overload, structural changes, and chronic myalgia [32,33,35].

Biopsies from chronically painful trapezius muscles revealed a higher proportion of hypertrophied Type I megafibers with poor capillarization compared with healthy controls, suggesting maladaptive remodeling toward large, poorly perfused slow-twitch fibers with increased reliance on anaerobic metabolism [36]. These findings support a model in which prolonged exposure of a limited pool of low-threshold motor units to monotonous static loading produces fiber type-specific structural changes that may contribute to persistent muscle pain. Complementary evidence shows that muscle pain also alters neural control, decreasing firing rates of low-threshold motor units while increasing those of high-threshold units, indicating a differential adjustment of motor-unit recruitment in response to pain [37].

Muscle typology strongly influences responses to high-intensity and eccentric exercise. Fast-twitch fibers are often more susceptible to structural damage, with biomarkers indicating preferential disruption of fast-fiber sarcomeres after high-intensity concentric-eccentric tasks that mimic sprinting and jumping [38,39]. Additionally, it was suggested that in muscles dominated by slow-twitch fibers, the relatively fewer fast fibers may undergo repeated recruitment during eccentric bouts, rendering them especially vulnerable and linking baseline fast-fiber percentage to torque loss and recovery kinetics [38,39].

Isolated fiber experiments indicate that Type IIA fibers can show significant force loss after eccentric protocols, whereas Type I fibers in the same muscle may be relatively spared, further supporting the idea of fiber type-specific susceptibility. However, clinical and biochemical evidence adds further nuance: in mild (grade I) muscle lesions, circulating fast-myosin levels rise disproportionately compared with slow-myosin and blood creatine kinase (CK), suggesting preferential fast-fiber involvement, while more severe (grade II–III) injuries show elevated levels of both fast and slow myosin. These findings highlight that although fast fibers often exhibit greater vulnerability, slow-twitch fibers can also be compromised under certain conditions, underscoring the complexity of fiber type-dependent injury mechanisms [38,40].

Current training paradigms in sport and rehabilitation frequently apply “one-size-fits-all” programs despite clear interindividual differences in muscle typology and fiber type-dependent fatigue and recovery profiles. Evidence indicates that fast- and slow-dominant individuals respond differently to repeated eccentric loading, implying that individualized strength training protocols tailored to fiber type composition could optimize performance, reduce cumulative fatigue, and potentially lower injury and pain risk [11,39,41]. Moreover, fiber type-specific remodeling in chronic pain states, the impact of sex, age, and occupational loading, and the bidirectional relationships between muscle damage, regeneration, and persistent pain are not yet integrated into a coherent, clinically oriented framework. In fact, despite the high prevalence of musculoskeletal pain disorders, most rehabilitation models focus primarily on symptom reduction and functional restoration [42,43,44]. Incorporating a fiber type perspective may therefore provide a more mechanistic framework for understanding how chronic pain alters muscle structure and function and how targeted rehabilitation strategies could address these specific adaptations.

This narrative review synthesizes current evidence on muscle fiber types and muscle pain, with emphasis on their metabolic, mechanical, and regenerative properties, and examines how fiber type biology may inform individualized strategies for the prevention and management of chronic musculoskeletal pain and exercise-induced muscle damage. To support this objective, relevant human studies were identified through structured searches of PubMed/MEDLINE, Scopus, and Web of Science. Priority was given to high-quality studies addressing fiber type heterogeneity, susceptibility to mechanical loading or injury, and clinical correlates of muscle pain, with selected animal data consulted only when necessary for physiological context. This process yielded 27 human studies on muscle pain and muscle injury, which are summarized in Table 1 and Table 2.

## 2. Satellite Cells and Muscle Damage

Human studies investigating satellite cells commonly rely on muscle biopsy techniques combined with histological and immunohistochemistry analyses to assess cellular content, activation, and fiber type-specific responses. In patients who had previously been exposed to monotonous and repetitive work tasks, strength training led to a marked increase in satellite cell content. This increase amounted to approximately 65% in Type I fibers and 164% in Type II fibers, indicating a stronger regenerative response in fast-twitch fibers [25].

Muscle damage involved both fiber types, though patterns differed: structural damage was evident in Type I fibers of patients with monotonous and repetitive work tasks with trapezius myalgia (19% more of satellite cells per fiber than Type II fibers) [45], containing a significantly higher proportion of Type I megafibers (grossly hypertrophied Type I muscle fibers) with poor capillarization [36], while exercise-induced disruption indicated substantial Type II fiber involvement [39,62].

In eccentric and concentric-eccentric exercise, Type II fibers were damaged first, showing sarcomere disruption and reduced force production (about 8% decrease in Type IIA peak force), while Type I fibers were initially spared [62]. Although, in eccentric exercise-induced muscle damage, clear damage was seen in Type I fibers, yet CK levels imply that Type II fibers were also involved, likely to a comparable extent [65]. In summary, satellite cell responses and muscle damage patterns highlight fiber type differences. Type II fibers show strong regenerative activation but are highly susceptible to early disruption, whereas chronic occupational loading predominantly affects Type I fibers, leading to structural changes.

## 3. Symptom Duration and Fiber Composition

The relationship between symptom duration and lumbar muscle morphology in chronic low back pain (CLBP) suggests time-dependent but heterogeneous, remodeling of muscle fibers. Several consistent patterns and important contradictions emerge from available studies.

Longer symptom duration has been associated with a shift toward a more glycolytic profile, specifically an increased proportion of Type IIX fibers and a concurrent reduction in Type I fibers [55]. Prolonged pain has also been linked to the appearance of Type I megafibers, consistent with maladaptive structural remodeling over time [36]. However, overall cross-sectional muscle area (RCSA) does not consistently correlate with symptom duration [67], and preliminary data indicate that the presence of at least one cytochemical alteration in fiber architecture may be associated with longer pain duration, an observation that requires confirmation in longitudinal studies [55].

Type IIC fibers are normally rare and remain uncommon in CLBP. When present, Type IIC proportions tend to be higher in early-stage pain (<1 year) and lower in long-standing pain (>3 years), which is compatible with an early, transient phase of fiber type transition following symptom onset [53,55]. This temporal pattern of early transformation was also observed in chronic neck pain, where a higher proportion of transitional Type IIC fibers was found almost exclusively in patients with symptoms lasting less than 16 months. In contrast, patients experiencing pain for more than 20 months lacked Type IIC fibers [50].

Morphological and pathological changes in the lumbar multifidus have also been reported. Higher pain intensity and greater disability correlate with smaller Type I fibers in the lumbar region [52,67]. In patients with disc herniation undergoing microdiscectomy, the multifidus frequently contained a greater proportion of pathological fibers, including focal myofibrillar degeneration [52]. Fiber atrophy affects both Type I and Type II fibers but is often more pronounced in Type II fibers, with probable consequences for force generation. Importantly, fiber atrophy in these cohorts was linked to objective signs of nerve-root involvement rather than to symptom duration, implying that denervation and ischemia, rather than simple deconditioning, may drive morphological deterioration in specific pathologies such as disc herniation [52,67].

Findings on fiber type distribution across studies are inconsistent. Some reports identify a reduction in Type IIB/IIX fibers and a relative shift toward a more oxidative phenotype (larger Type I RCSA and smaller Type IIB/IIX RCSA) [54], whereas others describe an increased proportion of Type II fibers, particularly Type IIB/IIX, together with reduced fiber diameters across both Type I and II fibers [51,53]. These discrepancies likely reflect differences in patient populations, symptom duration, underlying pathology (for example, nerve-root compression), and methodological approaches.

**Figure 2:**
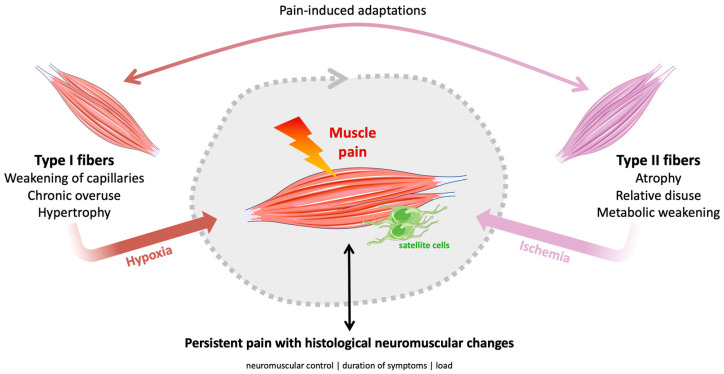
Conceptual model: chronic muscle pain according to the type of muscle fiber. Parts of this figure were drawn using pictures from Servier Medical Art by Servier (https://smart.servier.com, accessed on 5 January 2026), which is licensed under Attribution 4.0 International (Creative Commons CC BY 4.0).

In summary, chronic pain is associated with complex, time-dependent changes in fiber composition and morphology: early transitional changes (including transient Type IIC increases), later shifts in fiber type proportions, and pathology-specific atrophy driven in some cases by denervation/ischemia. In Figure 2, we propose a conceptual model related to CMP.

## 4. Muscle Fiber Size and Atrophy

Differences in muscle fiber size were investigated in patients with fibromyalgia (FM). No significant differences were observed in the overall proportion of Type I and Type II muscle fibers between FM patients and controls. However, patients with FM exhibited pronounced alterations in muscle fiber morphology, characterized by greater variability in fiber size and a disrupted fiber size distribution. Specifically, there was an increased prevalence of small-diameter fibers, particularly among Type II fibers, suggesting selective involvement or impaired maintenance of fast-twitch fibers. In addition to these structural changes, capillary density was reduced in individuals with FM, which may reflect compromised muscle perfusion and is likely to contribute to impaired muscle metabolism and reduced functional capacity [57]. Overall, FM preserves overall fiber type proportions but shows more small Type II fibers, greater fiber size variability, and reduced capillary density, indicating impaired muscle maintenance and perfusion.

## 5. Physical Activity, Occupation and Loading Patterns

The influence of physical activity level and occupational loading on multifidus muscle fiber characteristics has been investigated to better understand muscle adaptation to different functional demands. Overall, the level of physical activity did not appear to influence multifidus muscle fiber characteristics. Specifically, muscle fiber type composition and distribution did not differ significantly among individuals with high, medium, or low levels of physical activity [51].

In contrast, clear differences were observed in relation to occupational loading. Cleaners exhibited a smaller RCSA of Type II fibers compared with teachers, while no differences were found in Type I fibers. Similarly, female office workers with trapezius myalgia showed higher proportions of Type IIA fibers and lower proportions of Type I fibers compared to healthy controls [49]. These findings likely reflect distinct muscle activation patterns and mechanical demands associated with different occupational tasks, suggesting that sustained, occupation-specific loading may selectively influence fast-twitch muscle fiber morphology [32].

## 6. Sex and Age Differences

Sex- and age-related differences in muscle fiber characteristics have been examined in patients with chronic low back pain to better understand variability in muscle adaptation and pathology. In this context, men were found to exhibit a higher proportion of Type IIX fibers and a larger muscle fiber diameter compared with women [51], whereas women showed a greater proportion of Type I fibers [55].

Additionally, women demonstrated fewer Type IIC fibers and a higher prevalence of Type I megafibers, particularly in conditions such as trapezius myalgia. These alterations were often accompanied by poor capillarization, suggesting impaired muscle perfusion and potential metabolic consequences [36,55]. Conversely, men with work-related trapezius myalgia exhibit a different morphological response, characterized by Type IIA fiber hypertrophy accompanied by a proportional increase in capillary supply, as well as histological signs of an active muscle injury-regeneration cycle [48]. Furthermore, during experimentally induced trapezius pain, women showed a less effective protective decrease in muscle activity and lacked the adaptive recruitment of additional motor units seen in men during sustained painful contractions [68].

Age was not found to exert a significant influence on muscle fiber characteristics in patients with chronic low back pain. However, there was a tendency for increasing age to be associated with smaller overall muscle fiber size, especially among Type II fibers, along with a shift toward higher proportions of Type I fibers and reduced proportions of Type IIX fibers. These trends may reflect age-related remodeling and a gradual transition toward a more oxidative muscle phenotype [55].

Sex-related differences in muscle fiber size appear to develop during the transition from childhood to adulthood, and particularly during adolescence, associated with hormonal changes. In adults, males typically show larger cross-sectional areas in fast Type II fibers, particularly Type IIA, relative to Type I fibers, a pattern not observed in children. Much of the variation in Type II fiber size is explained by body weight and sex, whereas Type I fiber size is primarily related to body weight alone. These findings suggest that sex-specific muscle characteristics emerge with maturation, likely reflecting combined effects of growth, physical activity patterns, and hormonal changes [69]. In summary, sex influences muscle morphology: men tend to show a higher proportion of Type II fibers and larger fiber diameters, whereas women show more Type I fibers and a higher prevalence of Type I megafibers with poorer capillarization. Age exerts mild effects, generally trending toward smaller fibers and a more oxidative profile.

## 7. Regional Differences

Muscle fiber composition and motor unit recruitment patterns have been investigated in relation to muscle depth and pain-related motor control alterations. The deep layers of the multifidus muscle were found to be predominantly composed of Type I fibers compared with the more superficial layers. However, despite this structural distinction, no evidence of differential muscle fiber recruitment was observed during trunk extension tasks performed with or without experimentally induced pain [60]. Moreover, morphological investigations in patients with chronic lumbar spine pathology demonstrate that the superficial and deep regions of the multifidus undergo similar structural alterations, with no significant differences in fiber type composition, fiber cross-sectional area, or the extent of muscle degeneration between layers. Both regions appear to be equally affected by the pathological process [56].

In contrast, functional alterations in motor unit behavior were evident during repeated eccentric exercise bouts, where reduced activation of fast-twitch motor units was observed. This pattern may reflect the presence of an adaptive inhibitory mechanism, potentially serving to protect already stressed or damaged muscle tissue from further mechanical strain and injury [64].

The etiology of paraspinal muscle dysfunction appears to be related more to acquired adaptations than to an inherent or constitutionally adverse muscle fiber type profile. Specifically, no abnormalities have been identified in paraspinal muscle fiber size, fiber type composition, or overall fiber content that would suggest a primary structural predisposition [61].

Instead, evidence points toward activity- and load-related changes affecting muscle fiber integrity. Early overload and damage to Type I fibers were accompanied by an increase in satellite cell content and myonuclei, indicative of an active regenerative response. In contrast, Type II fibers exhibited reduced satellite cell content and greater degrees of atrophy, likely reflecting relative underuse or altered recruitment patterns [45]. Moreover, the absence of α-actinin-3 was associated with increased susceptibility to damage in Type II fibers, highlighting a potential genetic influence on fiber-specific vulnerability [63].

Supporting the concept of acquired pathology, a case series report described multifocal myofibrillar disorganization selectively affecting Type II fibers, further underscoring the role of secondary, load-dependent mechanisms in the development of paraspinal muscle dysfunction [59]. In this context, muscle dysfunction appears driven by acquired, load-related changes rather than inherent fiber type differences, with protective reductions in fast-twitch motor unit activation during repeated eccentric tasks and greater vulnerability of Type II fibers to damage.

## 8. Lean Body Mass

The relationship between muscle fiber size and body composition has been examined to better understand determinants of muscle morphology. Muscle fiber diameter was found to be strongly correlated with lean body mass, particularly for Type II fibers [42]. In line with these findings, lean body mass showed a significant positive correlation with the size of Type IIA and Type IIX fibers [55], suggesting that greater muscle mass is primarily associated with hypertrophy of fast-twitch and intermediate fiber types.

Moreover, these correlations help explain sex-related differences in fiber size, as males typically have greater lean mass and correspondingly larger Type II fibers than females, reinforcing the role of both body composition and sex in shaping muscle morphology [69].

A summary of the key differences among muscle fiber types is presented in Table 3.

## 9. Discussion

The present findings demonstrate that chronic musculoskeletal pain (CMP) is associated with fiber type-specific alterations in skeletal muscle morphology and regenerative behavior, rather than reflecting a constitutionally predetermined fiber type profile. This response accompanied by greater damage and atrophy in fast-twitch fibers, the presence of hypertrophied yet poorly capillarized Type I megafibers linked to chronicity, and the rare, transient appearance of Type IIC fibers together suggest distinct adaptive strategies to chronic overload and functional alterations.

The increase in satellite cell content following specific strength training, more than twofold greater in Type II than in Type I fibers, indicates that the trapezius muscle affected by chronic myalgia retains a substantial regenerative potential [25,70]. This is especially relevant because, prior to intervention, patients with chronic pain frequently exhibit a selective satellite cell deficit in Type II fibers [45], a pattern that resembles accelerated muscle aging [71]. The aging process is typically characterized by a preferential shift from fast-twitch to slow-twitch fibers, a reduction in Type II fiber-associated satellite cell pools, and progressive intramuscular fat infiltration, processes that may be further exacerbated by metabolic dysregulation and insulin resistance promoting ectopic lipid accumulation and impaired muscle glucose metabolism [72,73,74]. The pronounced satellite cells expansion in fast-twitch fibers after training, even in elderly (and probably in sedentary, overweight/obese subjects), suggests that targeted loading can partially reverse this imbalance and restore the myogenic capacity of these fibers [24,25]. This finding challenges the hypothesis that chronically overloaded Type I fibers in occupational settings become refractory to further myogenic stimuli.

Skeletal muscle is a highly plastic tissue that adapts to contractile demand and environmental conditions, resulting in coordinated changes in metabolism, contractile properties, and fiber type composition. Endurance exercise, largely via activation of the transcriptional coactivator peroxisome proliferator-activated receptor γ coactivator 1α (PGC-1α), promotes oxidative metabolism, shifts fibers toward fatigue-resistant phenotypes, enhances vascularization, and protects against muscle atrophy. Beyond metabolic remodeling, exercise and PGC-1α also modulate the muscle stem cell niche and immune environment, influencing satellite cell activity and macrophage polarization in ways that support muscle maintenance and regeneration. Importantly, the observed expansion of satellite cells and concomitant increase in macrophage content occur in the absence of classical markers of muscle damage or regeneration (e.g., centralized nuclei or embryonic myosin) [58], indicating that these cellular changes represent a beneficial physiological adaptation and pre-conditioning of muscle rather than a repair response to exercise-induced injury. This adaptive response was also associated with reductions in clinical pain, supporting a link between improved regenerative capacity and symptom improvement [16,75].

These findings align with growing evidence that exercise-induced cellular responses can foster a regenerative immune environment without provoking maladaptive tissue damage. Acute exercise, particularly when involving high mechanical strain, transiently recruits immune cells such as neutrophils, macrophages, and lymphocytes to muscle, where coordinated inflammatory signaling supports debris clearance, resolution of inflammation, and tissue repair. A timely transition of macrophages from pro-inflammatory to anti-inflammatory and pro-regenerative phenotypes is critical for effective regeneration and prevention of fibrosis, whereas persistent or dysregulated inflammation contributes to muscle degeneration and is implicated in conditions such as sarcopenia, insulin resistance, and muscular dystrophies. Regular moderate exercise appears to modulate immune cell infiltration and polarization in a manner that enhances repair while limiting chronic inflammation. Together, these observations suggest that when exercise elicits controlled satellite cell and immune cell activation without overt muscle damage, it supports muscle regeneration, functional improvement, and symptom relief, underscoring the importance of context-specific, well-regulated exercise interventions in chronic muscle disorders [17,76].

In patients performing monotonous and repetitive tasks, namely in the work context, greater satellite cells content in Type I fibers compared with Type II fibers [45] supports the Cinderella hypothesis, which proposes that low-threshold motor units are continuously recruited and subjected to chronic metabolic stress. This persistent activation may stimulate satellite cells expansion and myonuclear accretion to sustain protein turnover. In the myonuclear accretion phenomenon satellite cells proliferate and can fuse with existing fibers, contributing additional nuclei, which supports fiber repair and hypertrophy [77,78].

The presence of Type I megafibers in the same individuals [36] further suggests that these fibers belong to chronically activated motor units. On the other hand, experimental and modeling data suggest that motor units in postural muscles are recruited in rotation rather than in a fixed hierarchical manner, which would theoretically prevent the formation of permanently overloaded muscular units. Within this framework, factors that predispose to myofascial pain, such as increased muscle load or reduced muscle strength, can shorten motor unit relaxation periods and limit energetic recovery despite rotational recruitment. These conditions may impair the normal cycling of activation and deactivation that allows fibers to share mechanical demands. As a result, sustained tasks may lead to inefficient load distribution and accelerated fatigue. Thus, even in the absence of continuously active motor units, insufficient relaxation time under sustained load may still drive an energy crisis, providing an alternative mechanism by which chronic low-level activity can induce metabolic stress and muscle pathology [79,80]. Recent evidence further refines this concept by linking sustained low-level muscular activity to a cascade of metabolic and nociceptive events that promote peripheral sensitization. Prolonged or repetitive contraction can impair local perfusion, leading to relative ischemia, ATP depletion, mitochondrial dysfunction, and accumulation of metabolites such as lactate, protons, and inflammatory mediators. These metabolic disturbances activate and sensitize group III and IV muscle afferents nerve fibers, which in turn enhance nociceptive signaling and promote neurogenic inflammation. The resulting microenvironment favors persistent nociceptor activation, altered ion channel function, and local release of pain-evoking substances, creating a self-sustaining cycle of metabolic stress and peripheral sensitization [80,81,82,83]. In this context, muscle pain associated with repetitive low-load activity is not solely explained by permanent recruitment of specific motor units, but rather by the interaction between insufficient energetic recovery, microcirculatory compromise, inflammatory signaling, and nociceptor sensitization. Consequently, this framework integrates motor unit recruitment patterns with metabolic vulnerability and peripheral neural sensitization, offering a more comprehensive explanation for how chronic low-intensity muscle activity may evolve into persistent myofascial pain states [80,84,85,86].

Experimental models of exercise-induced muscle damage indicate preferential involvement of Type II fibers during eccentric and combined concentric-eccentric contractions, as reflected by early force loss and structural disruption [62,64,87]. This vulnerability likely arises from the lower intrinsic resistance of fast-twitch fibers to eccentric strain. In individuals with a predominantly Type I fiber composition, repeated recruitment of a limited pool of Type II fibers during initial exercise bouts may exacerbate damage in these fibers and contribute to torque loss [12,39]. Nevertheless, Type I fibers are not exempt from damage, as an exercise performed to task failure, regardless of load magnitude or repetition duration, ultimately necessitates recruitment of both Type I and Type II motor units. Consequently, when contractions are continued to volitional fatigue, metabolic stress and fiber activation extend across the full motor unit pool, making both fiber types susceptible to exercise-induced strain and contributing to early functional impairment during unaccustomed training [66].

In eccentric exercise, clear structural alterations have been observed in Type I fibers, while creatine kinase (CK) levels imply that Type II fibers are similarly involved [65]. This suggests that eccentric-induced muscle stress is not restricted to a single fiber type, but is mediated through multiple pathways, including excitation-contraction coupling disturbances, increased sarcolemma permeability, and extracellular matrix disruption. Apart from CK, these processes are accompanied by the plasma release of inflammatory and nociceptive mediators such as bradykinin, interleukins, tumor necrosis factor, nerve growth factor, and glial cell line-derived neurotrophic factor, as well as elevated reactive oxygen and nitrogen species. Together, these molecular responses contribute to delayed-onset muscle soreness, mechanical hyperalgesia, and transient impairments in muscle function following eccentric loading. These findings indicate that susceptibility to eccentric-induced muscle stress is not governed by a fixed fiber type hierarchy, but rather is context-dependent and shaped by contraction mode, motor unit recruitment strategies, mechanical loading characteristics, and baseline muscle fiber composition [18,88].

A study using multivariate models indicated that, with age and influenced by sex, symptom duration accounted for nearly 30% of the variance in fiber type distribution, with longer duration associated with a shift toward a more glycolytic profile, namely, higher proportions of fast-fatigable Type IIX fibers while Type I fibers decrease. Notably, symptom duration did not influence fiber size. Gross trunk muscle relative cross-sectional area (RCSA) correlated positively with lean body mass and negatively with age but showed no relationship with symptom duration. Cytochemical architectural abnormalities were more common in older patients and were independent of pain duration [55]. Collectively, these findings indicate that long-term adaptations in chronic low back pain primarily involve fiber type transitions rather than fiber atrophy, consistent with increased muscle fatigability and underscoring the importance of early active interventions [53,55,89].

Sex and age significantly influence fiber type distribution in chronic low back pain. Men exhibit a higher proportion of Type IIX fibers than women, largely attributable to differences in lean body mass, which account for up to 64% of variance in trunk muscle RCSA [55,69]. In parallel, aging is typically characterized by reductions in Type II fibers, associated satellite cell pools, and progressive intramuscular fat infiltration, changes that are further exacerbated by systemic metabolic dysregulation, particularly insulin resistance, which promotes ectopic lipid accumulation and impaired muscle glucose metabolism [72,73,74]. These aging-related alterations share several features with the muscle changes observed in chronic pain, including preferential Type II fiber atrophy, diminished Type II associated satellite cell content, and increased intramuscular fat. However, key distinctions remain: whereas aging reflects global, systemic physiological processes, CMP-related adaptations appear to arise primarily from localized overload, underuse, or altered fast-twitch motor-unit recruitment rather than from systemic metabolic or endocrine drivers. In CMP, these changes are more strongly linked to task-specific mechanical stress, reduced activation of fast-twitch motor units, and fiber-specific vulnerability (for example, in individuals lacking α-actinin-3), underscoring that the pathology is primarily acquired and load-dependent rather than constitutional [90,91,92,93].

In addition, sex-related differences in muscle fiber characteristics emerge during maturation, with adult males typically exhibiting larger cross-sectional areas in fast Type II fibers compared with Type I fibers, a pattern not observed in females or children and largely explained by differences in body mass and sex-related factors. Transitional fibers (Type IIC) are expected to be more prevalent in men, although their absolute proportion remains low, reinforcing the interpretation that fiber type adaptation in chronic low back pain is slow and cumulative rather than acute [55,69]. While normal aging reduces Type IIX fibers [94], prolonged chronic low back pain appears to override this tendency, promoting a relative increase in glycolytic fibers [55]. Thus, chronic pain may mask, or reverse expected age-related shifts in fiber type composition.

Interestingly, these sex-specific structural adaptations extend beyond the lumbar region and appear to be muscle-specific, as evidenced in the trapezius. While women with work-related trapezius myalgia tend to develop Type I megafibers with deficient capillarization, men exhibit a completely different morphological response, developing Type IIA fiber hypertrophy accompanied by a proportionally matched capillary network [48]. This divergence is not merely structural but also functional. A study demonstrated that during experimentally induced pain, women lack the protective neuromuscular adaptation seen in men, who recruit additional motor units to cope with fatigue. Consequently, this less efficient motor control, combined with the female prevalence of poorly capillarized Type I megafibers, further explains women’s higher vulnerability to work-related myalgia compared to the more robust compensatory mechanisms observed in men [68].

The scarcity of transitional Type IIC fibers further implies that fiber type conversion occurs slowly and continuously or that biopsies capture a stabilized post-transformation state rather than an acute remodeling phase [53,55,69]. This concept of a stabilized post-transformation state is strongly corroborated by findings in the cervical spine. Active fiber transformation occurs mainly during the first 2 years of neck pain. After this initial phase, the neck muscles return to a structurally stable condition, ceasing further fiber transformation despite the continuation of cervical dysfunction and chronic pain [50]. Importantly, while chronicity influences fiber type proportions, it does not determine fiber size, which is primarily dependent on lean mass. Highly oxidative fibers tend to remain smaller despite a high capacity for protein synthesis, due to competing signaling demands between myofibrillar protein accretion and mitochondrial biogenesis, as well as higher proteasome-mediated protein degradation. Thus, fiber size appears to be regulated predominantly by systemic factors such as mechanical loading and lean body mass, rather than by symptom duration or pain chronicity [55,95]. Moreover, degenerative alterations in internal fiber architecture appear to be more strongly associated with biological aging than with pain duration *per se* [53,55].

Consistent with this, Zhao et al. [67] found no relationship between symptom duration and multifidus RCSA but demonstrated that fiber atrophy was linked to objective signs of nerve root involvement. This suggests that denervation and ischemia, rather than cumulative deconditioning alone, drive morphological deterioration in specific pathologies such as disc herniation [52,67]. Patients with smaller Type I fibers exhibited greater pain and disability, supporting the notion that atrophy of tonic stabilizing fibers compromises segmental control of the spine [96]. In trapezius myalgia, selective hypertrophy of Type I fibers (megafibers) has been reported in women, consistent with chronic activation of low-threshold motor units [36]. However, this hypertrophy is not accompanied by proportional angiogenesis, resulting in deficient capillarization. The mismatch between fiber volume and oxygen supply may promote local hypoxia and anaerobic metabolism, favoring the accumulation of nociceptive metabolites and perpetuation of chronic pain [97]. However, this microcirculatory impairment and metabolic dysfunction appear to be reversible. Both strength and endurance training can significantly increase the number of capillaries around Type I and IIA fibers in myalgic trapezius muscles. By improving the capillary supply, these interventions enhance oxygen delivery to the working muscle fibers, effectively mitigating the local energy crisis and contributing to significant pain relief [46].

Comparisons across studies reveal conflicting patterns of fiber type transformation in low back pain [98]. Earlier studies reported a shift toward a glycolytic phenotype with increased Type II fibers and pronounced atrophy [51,53,67], whereas more recent work has shown preservation or even predominance of Type I fibers with reductions in Type IIX fibers [54]. These discrepancies can be explained by differences in pain etiology and adaptation mechanisms. First, clinical populations differ substantially. Studies reporting glycolytic shifts largely investigated patients with structural pathology (disc herniation, stenosis, scoliosis), where denervation and ischemia directly influence fiber morphology [67]. Supporting this distinction, in patients undergoing surgery for severe structural pathologies (such as stenosis and herniation), the multifidus muscle exhibits profound global degeneration, elevated fibrosis, and persistent signs of muscle damage that outpace regenerative capacity. This reinforces that nerve compression and structural severity drive morphological deterioration [56]. In contrast, investigations of non-specific chronic low back pain [54] reflect adaptations in the absence of nerve compression. Second, competing adaptation mechanisms may operate: classic disuse favors fast, fatigable fibers [51], whereas altered neuromuscular control in non-specific chronic low back pain may produce tonic, low-frequency activation patterns [99] that induce slow fiber transformation (IIX > IIA > I) [100]. Thus, muscle remodeling appears contingent on both clinical subgroup and motor control strategy.

Deep multifidus fibers were found to be predominantly Type I compared with superficial layers, reflecting their postural function. However, no consistent differences in recruitment have been observed during trunk extension with or without experimentally induced pain. Evidence from eccentric exercise suggests preferential recruitment and disruption of Type II fibers [62,87], and reduced fast motor unit recruitment during repeated bouts may represent an adaptive inhibitory mechanism that protects previously damaged fibers [64]. On the other hand, the clinical hypothesis that the deep multifidus is uniquely susceptible to structural degeneration in chronic pain conditions is not supported by other studies. A study in patients with chronic lumbar spine pathology demonstrated that degenerative changes, including fiber atrophy, shifts in fiber type composition, and fat infiltration, occur to a similar extent in both the superficial and deep regions, rather than selectively affecting the deep layer [56]. Interestingly, this lack of motor control adaptation in the lumbar region contrasts with findings in the cervical region. When experimental pain was induced in the upper trapezius, individuals (particularly men) effectively altered their motor strategy by recruiting additional motor units to cope with fatigue. This discrepancy suggests that neuromuscular adaptations to acute pain are highly region-specific, depending heavily on the biomechanical role of the muscle and the individual’s sex [68].

Muscle morphology is sensitive to occupational demands. Cleaning workers exhibit smaller Type II fibers RCSA compared with teachers, without changes in Type I fibers, indicating that static and repetitive low-force tasks selectively fail to preserve fast-twitch fiber size [32]. This adaptation is also evident in sedentary professions. It was observed that office workers with trapezius myalgia shift toward a faster, glycolytic profile (increased Type IIA and decreased Type I fibers), likely a metabolic adaptation to local hypoxia caused by prolonged static computer work. Furthermore, both myalgic and healthy workers exhibited enlarged Type I fibers, strongly supporting the Cinderella Hypothesis which states that continuous low-level tasks chronically overload low-threshold motor units [49]. In contrast, overall physical activity level does not appear to influence fiber type distribution or diameter in multifidus muscles [51]. This suggests that generalized activity is insufficient to counteract pain-related inhibition and abnormal motor control, which may perpetuate histological damage through reduced reflex activation and abnormal loading patterns [101,102,103].

Genetic variation in α-actinin-3 influences fiber type-specific responses to eccentric loading. Individuals expressing α-actinin-3 (recessive-recessive genotype) exhibit increased stiffness specifically in Type IIA fibers after eccentric exercise, potentially conferring Z-disc protection [104]. This viscoelastic adaptation may reduce sarcomere overstretch and limit structural disruption in subsequent exposures [62]. However, protective effects appear modest and context-dependent [63]. In fact, genetic variability may influence individual differences in muscle fiber composition, regenerative potential, and susceptibility to musculoskeletal pain [105,106,107]. Although the genetic contribution to chronic musculoskeletal pain remains incompletely understood, future studies integrating genetic and molecular analyses may provide further insight into interindividual variability in muscle remodeling and recovery.

In FM, most muscle biopsy studies do not demonstrate consistent differences in overall muscle fiber type proportions when compared with healthy controls [108]. It is possible that the increased heterogeneity in muscle fiber morphology is characterized by marked variability in fiber size and a higher prevalence of small-diameter fibers, particularly among Type II fibers [57]. These alterations probably occur in the absence of overt inflammatory changes and are not specific enough to define a primary myopathic process. The selective involvement of Type II fibers, together with reports of reduced capillary density and microvascular abnormalities, may nevertheless compromise oxygen delivery and metabolite clearance during muscle activity. Such peripheral alterations could contribute to post-exercise fatigue, pain, and exercise intolerance in FM, even though they are likely secondary and interact with central pain-processing mechanisms rather than constituting a primary cause of the syndrome. Thus, these peripheral muscle alterations are best interpreted within a broader pathophysiological context in which FM is conceptualized as a centralized pain syndrome, driven by dysregulation of central nervous system processing, impaired descending pain modulation, neuroinflammation, and neurotransmitter imbalances. Accordingly, muscle changes are likely secondary or contributory factors that interact with central sensitization mechanisms, rather than constituting a primary myopathic cause of the disorder [109].

Taken together, these findings indicate that muscle dysfunction in chronic pain does not arise from an inherently adverse fiber type profile but from acquired, fiber-specific maladaptations. Type I fibers exhibit features of chronic overload and compensatory satellite cells expansion, whereas Type II fibers display patterns consistent with disuse, satellite cells depletion, and atrophy, resembling premature muscle aging. This remodeling can occur without marked inflammatory activation, suggesting a chronic homeostatic rather than acute injury response. Therefore, rehabilitation strategies should move beyond nonspecific activity and incorporate targeted loading of fast-twitch fibers to reverse atrophy and fatigability, while simultaneously promoting oxidative capacity to improve endurance and metabolic efficiency. In humans, where muscle fiber types exhibit relatively similar cross-sectional areas, interventions that enhance oxidative adaptations, through appropriately dosed endurance training, combined modalities, or adjunct nutritional and pharmacological strategies, may improve fatigue resistance without compromising muscle mass. Importantly, preservation and reconditioning of Type II fibers remain essential for maintaining strength, power, and functional capacity, underscoring the need for balanced, fiber type-specific rehabilitation programs that support both metabolic health and neuromuscular performance [110,111,112,113].

Clinical interventions should extend beyond generic strengthening and flexibility programs to include strategies that specifically target fast-twitch fibers, counteracting Type II atrophy and restoring force-generating capacity, while also enhancing oxidative metabolism and perfusion in slow-twitch fibers—particularly in muscles subjected to sustained low-level activation. Additionally, interventions should incorporate exercise modalities that stimulate satellite cell activity, promoting adaptive regeneration and long-term muscle resilience [64,114,115]. In FM, combined aerobic and resistance training may be required to address both reduced capillarization and fiber size heterogeneity. This combined approach appears to be the most effective for improving quality of life, alleviating pain, and enhancing physical function in individuals with the disease. Supporting this, a network meta-analysis of 51 RCTs gathering 2873 women with FM found that aquatic exercise was most effective in reducing pain in the short term, while resistance training showed the greatest benefits in the long term. These findings highlight the importance of incorporating both aerobic and resistance modalities to achieve sustained therapeutic effects in FM [114,116]. In fact, it is suggested that progressive resistance training performed two to three times per week at moderate-to-high intensity is particularly effective in stimulating hypertrophy and functional recovery of Type II fibers. In contrast, aerobic training at moderate intensity appears to primarily support oxidative capacity and capillarization of Type I fibers [117,118,119,120,121].

It has also been demonstrated that active rehabilitation strategies are effective in the management of chronic musculoskeletal pain. There are superior clinical outcomes with predominantly active physiotherapy approaches compared with passive or combined modalities in chronic low back pain. These findings are consistent with the concept that mechanical loading and muscle activation are essential stimuli for restoring muscle fiber function and reversing disuse-related adaptations [42,122,123,124].

The heterogeneity of findings across studies likely reflects methodological and clinical variability. Differences in patient populations, symptom duration, occupational loading patterns, and biopsy location may significantly influence fiber type composition. In addition, methodological factors such as fiber-typing techniques, immunohistochemistry protocols, and sample size may contribute to inconsistent observations. Finally, chronic musculoskeletal pain represents a heterogeneous group of conditions with distinct pathophysiological mechanisms, which may further explain the variability reported in the literature.

Future research should prioritize translational models that integrate detailed muscle histology with functional neuromuscular assessments, including high-density surface electromyography, in vivo imaging of muscle quality, and reflex pathway evaluation during dynamic tasks. Incorporating artificial intelligence and machine learning approaches could further enhance data analysis, enabling real-time prediction of fiber type recruitment, personalized exercise prescription, and identification of the most effective interventions to optimize muscle adaptation and regeneration [99,125,126,127,128]. Longitudinal studies stratifying patients by motor control strategy, pain phenotype, and treatment response are needed to identify subgroups with differential muscle adaptation trajectories. Incorporation of molecular biomarkers of regeneration, including satellite cell activity and growth factor signaling, may further clarify mechanisms of muscle plasticity in chronic pain. Ultimately, pragmatic clinical trials should evaluate whether interventions tailored to fiber type-specific vulnerabilities (e.g., fast-fiber-targeted strength training versus oxidative conditioning of postural muscles) improve patient-centered outcomes such as function, work ability, and quality of life.

## 10. Conclusions

Skeletal muscle exhibits remarkable plasticity, with fiber type composition, metabolic profile, and regenerative capacity shaped by age, sex, physical activity, and pathological conditions. In chronic musculoskeletal disorders, including chronic low back pain and fibromyalgia, maladaptive changes, such as Type II fiber atrophy, reduced oxidative capacity, altered perfusion, and impaired satellite cell function, contribute to diminished force production, increased fatigability, and functional decline. Exercise interventions, particularly those combining aerobic and resistance modalities, can counteract these changes by promoting fast-twitch fiber recruitment, enhancing oxidative metabolism in Type I fibers, and stimulating satellite cell-mediated regeneration. Translational evidence highlights that these adaptations occur even in the absence of overt muscle damage, supporting the concept of exercise-induced pre-conditioning for improved regenerative capacity. Sex- and age-related differences, along with lean body mass, modulate these responses, emphasizing the need for individualized, context-specific exercise strategies to restore muscle structure, function, and regenerative potential in chronic musculoskeletal conditions. Integrating molecular profiling of satellite cells and muscle fibers may help clarify the mechanisms underlying impaired regeneration and fiber type remodeling.

From a clinical perspective, these findings support the development of individualized rehabilitation programs that specifically target fiber type deficits, combining resistance training to restore Type II fiber function with endurance-based strategies to optimize oxidative capacity and microvascular adaptations.

## Figures and Tables

**Table 1:** Summary of relevant references concerning the relationship between muscle fiber types and muscle pain studies in humans.

Reference/Type	Population	Type of Pain	Evaluation Method	Results
[25] Randomized controlled trial	42 women with trapezius myalgia: monotonous and repetitive work tasks	Chronic trapezius myalgia	Immunohistochemistry	Type I fibers: increase of 65% in satellite cells; Type II fibers: increase of 164% in satellite cells
[45] Experimental, cross-sectional	62 female office workers: 42 with myalgia in trapezius muscle, with monotonous and repetitive work tasks	Chronic trapezius myalgia	Fluorescent laminin staining	Type I fibers contained more satellite cells than Type II fibers; Type II fibers, less frequently recruited during sustained low-intensity activity, had fewer satellite cells and showed smaller fiber areas indicative of atrophy
[46] Randomized controlled trial	21 women with work-related neck and shoulder myalgia divided into three 10-week training programs	Chronic Trapezius myalgia	Enzyme-immunohistochemical analysis	Strength training increased Type IIA area (+49%) and proportion Coordination decreased Type I area; there was also an increased capillarization in Type I and IIA
[47] Case–control	10 female patients with chronic trapezius myalgia due static and repetitive work tasks and 5 healthy females	Chronic Trapezius myalgia	Enzyme histochemical, immunohistochemical and biochemical analyses	Patients had significantly larger Type I fibers and a lower capillary-to-fiber area ratio for Type I and Type IIA fibers
[36] Case–control study	42 female workers with trapezius myalgia and 20 female matched workers without	Trapezius myalgia	Histochemical	Significantly higher proportion of Type I megafibers in females with trapezius myalgia and poor capillarization
[32] Cross-sectional case–control study	25 female cleaners with trapezius myalgia and 25 without; 21 healthy female teachers without repetitive or static muscle work	Trapezius myalgia	Electromyogram and Immunohistochemistry	Smaller cross-sectional area of Type II fibers in cleaners compared to teachers; no difference in Type I fibers
[48] Case–control study	10 male forest machine operators with myalgia, 9 without myalgia, and 6 healthy controls	Trapezius myalgia	Histochemistry and immunohistochemistry	Men with myalgia showed significant Type IIA fiber hypertrophy with a proportionally matched increase in capillarization showing an active injury-regeneration cycle
[49] Cross-sectional study	17 female office workers with myalgia and 15 healthy controls	Trapezius myalgia	Major histocompatibility complex immunohistochemistry	Higher proportions of Type IIA and IIA/IIX fibers, and lower proportions of Type I fibers in the myalgia group No differences in fiber size between groups, but Type I fibers were significantly larger than Type II fibers in both groups
[50] Observational study	24 patients undergoing surgery for cervical dysfunction with severe neck pain	Chronic neck pain	Histochemical methods	Symptom duration < 16 months showed higher proportion of transitional Type IIC fibers (active transformation) Symptom duration > 20 months lacked Type IIC fibers
[51] Cross-sectional case–control study	64 patients with low back pain, 17 healthy control individuals	Low back pain	Histochemical	Low back pain showed a higher Type II fiber proportion The physical activity level did not influence fiber type and diameter
[52] Observational study	30 healthy adults (11 men) undergoing microdiscectomy for lumbar disc herniation	Low back pain	Immunohistochemistry and epifluorescence	Affected multifidus contained a higher proportion of pathological fibers Higher pain intensity and disability linked to smaller Type I fibers
[53] Cross-sectional controlled study	21 low back pain patients and 21 healthy controls	Low back pain	Histochemical	Patients had more Type IIB than Type I fibers; early-stage (<1 year) pain was linked to higher Type IIC proportions, while long-term (>3 years) pain showed marked reduction
[54] Cross sectional study	20 chronic low back pain and 18 healthy controls	Low back pain	Immunofluorescence	Pain is associated with fewer Type IIB fibers and a shift toward a more aerobic profile, with larger Type I and smaller Type IIB cross-sectional areas
[55] Experimental study	59 patients (30 women) with chronic low back pain	Chronic low back pain	Histochemical and magnetic resonance	Type I: Longer symptoms, lower proportion; higher in pathological biopsies; Female > Male Type IIA: Unaffected by symptom duration; diameter strongly tied to pain. Type IIX: Longer symptoms, higher proportion + stronger glycolytic profile; diameter correlates with pain; Male > Female Type IIC: Very low in chronic cases, Male > Female
[56] Observational study	16 patients undergoing lumbar spinal surgery	Chronic low back pain	Histochemical and MRI	No significant differences in fiber type distribution, size, fat infiltration, or muscle degeneration between the superficial and deep multifidus Chronic degeneration affects the multifidus globally
[57] Experimental Study	37 healthy postmenopausal women with (n = 14) and without (n = 23) Fibromyalgia (FM)	FM with a fatiguing exercise	Histochemistry, immunohistochemistry and electron microscopic	FM patients show similar Type I/II proportions, but greater fiber-size variability, more small fibers, and lower capillary density
[58] Case series	24-year-old man, 14-year-old male, and 47-year-old women	Myalgia	Histochemistry and immunohistochemistry	Type I fibers predominance with a selective uniform atrophy or a predominant multiple area of focal myofibrillar degeneration
[59] Case series	10-year-old girl, 22-year-old man, and 55-year-old man	Myalgia and cramps	Electromyogram, histochemistry, immunohistochemistry and electron microscopic	Acquired multifocal myofibrillar disorganization selectively affects Type II muscle fibers

Legend: FM, fibromyalgia.

**Table 2:** Summary of relevant references concerning the relationship between muscle fiber types and muscle induced injury studies in humans.

Reference/Type	Population	Type Muscle Injury	Evaluation Method	Results
[60] Cross-sectional experimental study	15 young males	Experimentally induced low back muscle pain with hypertonic saline solution	Magnetic resonance	Deep multifidus fibers are predominantly slow twitch compared to the superficial layer; no differential recruitment has been found following trunk extension with and without pain induction
[61] Investigative case–control study	35 males with chronic low back pain and 32 control	Excessive paraspinal muscle fatigue by exercise	Electromyography, histomorphometry and immunohistochemistry	Paraspinal muscle dysfunction did not stem from a constitutionally predetermined adverse fiber type profile
[39] Experimental research article	Nine healthy sedentary men not involved in strenuous eccentric exercise in past 6 months	Eccentric exercise induced muscle damage	Immunohistochemistry	Damage to Type II preceded damage to Type I fibers
[62] Experimental study	Eight non-athletic young men	Intensive eccentric knee flexion exercise	Muscle biopsies with suction, blood, and pain scores	Type IIA fibers exhibit an 8% drop in peak force 5 h post-eccentric exercise, with no changes in Type I fibers; their stiffness response is influenced by the ACTN3 R577X polymorphism
[63] Experimental study	20 healthy young men	Eccentric exercise	Blood creatine kinase	The absence of α-actinin-3 is associated with greater muscle damage in Type II fibers
[64] Case–control study	26 healthy male college students divide in two groups	Maximal eccentric exercise	Electromyographic activity	Reduced activation of fast-twitch motor units during the second eccentric bout
[65] Experimental study	Two groups of 11 healthy male volunteers	Eccentric exercise-induced muscle damage	CK, slow-twitch skeletal myosin heavy chains and cardiac troponin I	Clear damage was seen in Type I fibers, yet CK levels imply that Type II fibers were also involved, likely to a comparable extent
[38] Experimental study	10 healthy men, no myotendinous injuries and no training program	Exercise induced muscle damage	Blood sampling-ELISA	Concentric-eccentric inertial exercise: sarcomere disruption in Type II fibers, while Type I fibers remain unaffected
[66] Experimental study	10 recreationally trained young men	Resistance exercise	Biopsies (glycogen depletion in Type I and II fibers), EMG, and anabolic signaling markers	Type I and II fibers were similarly activated regardless of load or repetition duration when exercise was performed to task failure, indicating that full motor unit recruitment occurs independent of load magnitude

Legend: ACTN3, *α*-actinine-3 gene; CK, blood creatine kinase; ELISA, enzyme-linked immunosorbent assay; h, hour.

**Table 3:** Comparison of Fiber Types in Pain and Dysfunction.

Aspect	Type I (Slow Twitch) Fibers	Type II (Fast Twitch) Fibers (IIA/IIX)	Type IIC (Transitional) Fibers
Satellite cell response to strength training	↑ satellite cells	↑↑ satellite cells Stronger regenerative response	Can be influenced
Damage patterns	Common in repetitive work, hypertrophied megafibers with poor capillarization	Often damaged first in eccentric/concentric-eccentric exercise, sarcomere disruption and reduced force	May increase transiently
Symptoms (low back pain)	Lower proportion with longer symptoms; presence of Type I megafibers linked to chronicity	Longer symptoms linked to ↑ Type IIX and glycolytic profile; later shift sometimes toward more aerobic profile; contradictory findings across studies	Rare in chronic pain; higher proportions seen in early (<1 year) symptoms suggesting early fiber transformation
Fiber size and atrophy	Chronic pain associated with smaller Type I fibers	Atrophy often more pronounced; reduced strength potential; many studies report smaller Type II fibers	Not typically described as selectively atrophied
Physical activity influence	No clear differences across activity levels	No clear differences across activity levels	Not found
Sex differences	Women tend to have more Type I fibers; more megafibers in some pain conditions.	Men tend to have more Type IIX fibers and larger fiber diameter	No clear evidence, probably fewer in women
Age tendencies	Tendency toward higher proportion with aging	Aging tends to reduce fiber size and proportion, especially Type IIX	Not found

Legend: ↑, increased; ↑↑, highly increased.

## Data Availability

No new data were created or analyzed in this study.

## Abbreviations

The following abbreviations are used in this manuscript:

ACTN3	*α*-actinine-3 gene
ATP	adenosine triphosphate
ATPase	adenosine triphosphatase
CK	blood creatine kinase
CMP	chronic musculoskeletal pain
DAMPs	damage-associated molecular patterns
ELISA	enzyme-linked immunosorbent assay
FM	fibromyalgia
MHC	myosin heavy chain
MSD	musculoskeletal disorder
Pax7	pair box 7 protein
PGC-1α	peroxisome proliferator-activated receptor γ coactivator 1α
RCSA	relative cross-sectional area
